# Old Drugs, New Opportunities: Advancing Cancer Care Through Repurposing

**DOI:** 10.1002/ueg2.70184

**Published:** 2026-01-31

**Authors:** Norman R. Williams

**Affiliations:** ^1^ UCL Division of Surgery & Interventional Science London UK

Dear Editor,

Advances in cancer treatment continue to be made, driven by scientific innovation and an improved understanding of tumour biology. However, the development of new drugs has become increasingly expensive, and these escalating costs are reflected in the prices ultimately charged to health authorities and patients. Some modern treatments are now priced at more than $2 million USD per single dose, placing them well beyond the reach of most health systems and severely limiting patient access [1]. The widening gap between scientific potential and real‐world practice highlights the need for alternative strategies that can deliver effective treatments without imposing unsustainable financial burdens.

Drug repurposing (also known as repositioning) offers one such strategy. It involves identifying new therapeutic indications for medicines that have already been approved for other conditions. This approach provides several important advantages. Repurposed drugs come with a well‐established safety profile, substantially reducing the uncertainty addressed during early‐phase drug development. The drugs are often off‐patent, which means that cheaper generic versions are available. These factors make repurposing particularly attractive in oncology, where there is an increasing need for safe, affordable treatment options.

Nevertheless, drug repurposing is not without its challenges. Pharmaceutical companies (traditionally a main driver of drug trials) have little commercial incentive to invest in evaluating older off‐patent drugs. Furthermore, many repurposed medicines may offer only incremental therapeutic benefit, leading to scepticism even where the biological rationale is sound [2]. However, there are many examples demonstrating that repurposed therapies can be both effective and practice‐changing, including all‐trans retinoic acid (ATRA) and arsenic for acute promyelocytic leukaemia (APL), and thalidomide for multiple myeloma [3].

One widely studied group of drugs for repurposing are the *β*‐blockers. Developed for cardiovascular conditions such as hypertension and arrhythmias, accumulating evidence suggests that these agents may also exert anticancer effects. Several observational studies have reported associations between *β*‐blocker use and beneficial effects in several types of cancer [4, 5]. Importantly, not all *β*‐blockers appear to be equal in this regard. Propranolol, one of the earliest and most widely used *β*‐blockers, has distinctive properties that set it apart from other drugs in this class.

Notably, propranolol does not have particularly high *β*‐blocker activity (measured by β2‐and β1‐receptor affinity, binding duration, and efficacy) [6]. Propranolol does have a quite separate ability to trigger intracellular free calcium (Ca^2+^_i_) release, which provides a potential mechanistic explanation for anti‐cancer properties, including inhibition of migration, proliferation, and induction of apoptosis. In contrast, atenolol and metoprolol do not induce Ca^2+^i release and demonstrate little evidence of meaningful anticancer activity [7]. The calcium‐mobilising effects of propranolol are observed even in cells with minimal *β*‐adrenergic receptor expression, further underscoring that its anticancer potential likely extends beyond classical adrenergic pathways [8].

In this issue of UEGJ, new evidence from Emilsson et al. [9] provides additional evidence for use of *β*‐blockers in cancer treatment. Using real‐world data, the authors conducted an emulated target trial exploring whether *β*‐blocker use following the diagnosis of colorectal polyps could reduce subsequent colorectal cancer (CRC) incidence. The study population was large (30,399 individuals) and defined by straightforward eligibility criteria: adults aged 45–79 with a first colorectal polyp, no prior CRC or *β*‐blocker use, and no contraindications. Use of *β*‐blockers within 2 years of polyp diagnosis was associated with a lower incidence of CRC over a median follow‐up of 8 years. Sensitivity analysis restricted to propranolol users showed a similar reduction (HR ≈ 0.88).

Although the authors emphasise that this is the first emulated target trial assessing *β*‐blockers for CRC prevention, it is important to recognise the inherent limitations of observational designs such as this, including potential residual confounding and incomplete lifestyle data. Even so, the study demonstrates the value of modern analytical techniques applied to large registry‐based datasets. Similar approaches (e.g., the REVERSE model [10]) are increasingly being used across medicine to generate robust preliminary evidence prior to embarking on costly randomised trials.

An advantage of target trial emulation is that it allows detailed consideration of eligibility criteria, follow‐up schedules, exposure definitions, and outcome measures before undertaking a randomised controlled trial (RCT). Ultimately, however, RCT evidence remains the gold standard. If repurposed medicines like propranolol are to claim a place in routine oncology practice, their value must be confirmed in prospective, well‐designed trials. Such studies are particularly needed in resource‐poor settings, where access to new high‐cost cancer treatments is limited and where affordable repurposed therapies could have a profound impact.

Now is the time for such trials. Drug repurposing offers a pathway towards more equitable care. With coordinated effort, imaginative trial design, and commitment to accessible innovation, repurposed medicines may help bring life‐extending cancer treatments within reach for many more patients. Far from being a second‐best solution, repurposing represents an opportunity to deliver high‐value care that is both scientifically grounded and globally inclusive. It is time to explore this opportunity with optimism and determination.

## Funding

The author has nothing to report.

## Conflicts of Interest

The author declares no conflicts of interest.

## Data Availability

Data sharing not applicable to this article as no datasets were generated or analysed during the current study.
